# Prognostic Value of Coronary Artery Calcium Score in Hospitalized COVID-19 Patients

**DOI:** 10.3389/fcvm.2021.684528

**Published:** 2021-07-09

**Authors:** Maria-Luiza Luchian, Stijn Lochy, Andreea Motoc, Dries Belsack, Julien Magne, Bram Roosens, Johan de Mey, Kaoru Tanaka, Esther Scheirlynck, Sven Boeckstaens, Karen Van den Bussche, Tom De Potter, Berlinde von Kemp, Xavier Galloo, Clara François, Caroline Weytjens, Steven Droogmans, Bernard Cosyns

**Affiliations:** ^1^Department of Cardiology, University Hospital of Brussels (Centrum voor Hart-en Vaat ziekten, Universitair Ziekenhuis Brussel), Brussels, Belgium; ^2^Department of Radiology, University Hospital of Brussels, Brussels, Belgium; ^3^CHU Limoges, Hôpital Dupuytren, Service Cardiologie, Limoges, France; ^4^INSERM 1094, Faculté de médecine de Limoges, 2, rue Marcland, Limoges, France; ^5^Faculty of Medicine and Pharmacy, Vrije Universiteit Brussel, Brussels, Belgium

**Keywords:** Corona virus, coronary artery calcium score, major adverse cardiac and cerebral event, chest computed tomography, risk stratification

## Abstract

**Background:** The association of known cardiovascular risk factors with poor prognosis of coronavirus disease 2019 (COVID-19) has been recently emphasized. Coronary artery calcium (CAC) score is considered a risk modifier in the primary prevention of cardiovascular disease. We hypothesized that the absence of CAC might have an additional predictive value for an improved cardiovascular outcome of hospitalized COVID-19 patients.

**Materials and methods:** We prospectively included 310 consecutive hospitalized patients with COVID-19. Thirty patients with history of coronary artery disease were excluded. Chest computed tomography (CT) was performed in all patients. Demographics, medical history, clinical characteristics, laboratory findings, imaging data, in-hospital treatment, and outcomes were retrospectively analyzed. A composite endpoint of major adverse cardiovascular events (MACE) was defined.

**Results:** Two hundred eighty patients (63.2 ± 16.7 years old, 57.5% male) were included in the analysis. 46.7% patients had a CAC score of 0. MACE rate was 21.8% (61 patients). The absence of CAC was inversely associated with MACE (OR 0.209, 95% CI 0.052–0.833, *p* = 0.027), with a negative predictive value of 84.5%.

**Conclusion:** The absence of CAC had a high negative predictive value for MACE in patients hospitalized with COVID-19, even in the presence of cardiac risk factors. A semi-qualitative assessment of CAC is a simple, reproducible, and non-invasive measure that may be useful to identify COVID-19 patients at a low risk for developing cardiovascular complications.

## Introduction

Coronavirus disease 2019 (COVID-19) has significantly impacted the healthcare system, due to the rapid spread of infection and unpredictable disease course. Studies have shown that advanced age and comorbidities including hypertension, diabetes mellitus, cardiovascular diseases, and cerebrovascular diseases are predictors of an unfavorable prognosis and mortality in COVID-19 infection (1–4). Coronary artery calcium (CAC) score assessed by computed tomography (CT) is considered a risk modifier in primary prevention of cardiovascular disease (5, 6).

The CAC score offers two main assets: (1) it has an independent additional value in the prediction of all-cause mortality and mortality due to coronary artery disease in asymptomatic individuals; (2) it may reclassify patients considered as being at low or intermediate risk according to the clinical risk scores at high risk of atherosclerotic coronary events (6–9).

However, data regarding the role of CAC score in the prediction of cardiovascular events and outcome in COVID-19 patients are still scarce.

We hypothesized that the absence of CAC might have an additional predictive value for an improved cardiovascular outcome of hospitalized COVID-19 patients.

## Materials and Methods

We prospectively included 310 consecutive hospitalized patients with confirmed COVID-19 by real-time reverse transcription polymerase chain reaction (RT-PCR) test, between March 2020 and April 2020. Thirty patients with a history of coronary artery disease (stable angina, unstable angina, history of acute coronary syndrome) were excluded from the analysis. Demographics, medical history, clinical characteristics, laboratory findings, imaging data, in-hospital treatment, and outcomes were retrospectively analyzed. A composite endpoint [major adverse cardiovascular events (MACE)] was defined as all-cause mortality, heart failure, acute coronary syndrome, atrial fibrillation, and stroke.

**Figure 1:**
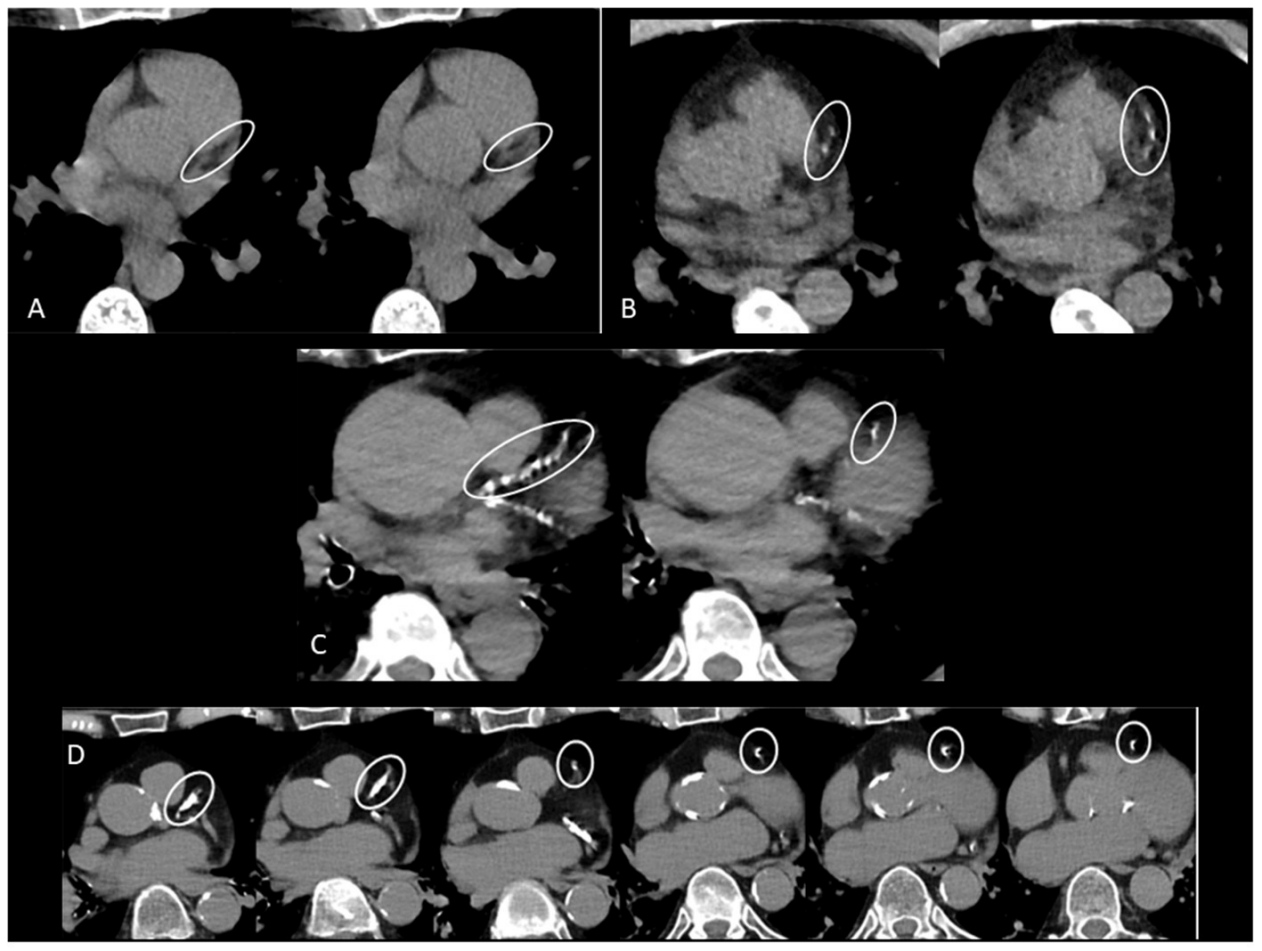
**(A)** CAC score zero in left anterior descending (LAD) coronary artery. **(B)** Mild CAC in LAD. **(C)** Moderate CAC in LAD. **(D)** Severe CAC in LAD.

In the absence of widely available RT-PCR at the beginning of the pandemic, chest CT had been systematically performed in all suspected COVID-19 patients. All patients were scanned on an Apex Revolution CT (GE Healthcare, Milwaukee, WI, USA). The low-dose non-contrast CT thorax scan protocol consisted of a 128 × 0.625 mm spiral acquisition with pitch 1, rotation time 0.35 s, automated kVp selection and automated mA modulation. Images with 1.25 mm slice thickness were reconstructed with deep learning image reconstruction (DLIR) set at medium level. The average volume CT dose index (CTDIvol) and dose-length product (DLP) were 4.4 mGy (95% CI: 4.3–4.5) and 159 mGy·cm (95% CI: 157–162), respectively. Visual assessment of CAC was performed using ordinal scoring: each of the four main coronary arteries was identified (left main, left anterior descending, left circumflex, and right coronary artery). Calcium was scored as 0, 1, 2, or 3 for every artery, corresponding to absent, mild, moderate, or severe CAC. Mild CAC was defined as involvement of less than one third of the vessel length, moderate as involvement of one to two thirds of the vessel length and severe CAC as involvement of more than two thirds of the vessel length. A total score was calculated by summing the score of each vessel. The total score was then categorized as 0 (undetectable), 1–3 (mild), 4–5 (moderate), and ≥ 6 (severe) (10) (Figure 1).

Intraobserver and interobserver reproducibility analyses of CAC score were performed by repeating the measurements in 20 random patients by the same primary investigator 2 weeks after the first assessment and by an additional investigator, respectively. During the repeated analysis, the investigators were blinded to any previous results.

The study was approved by the local Ethical Committee of the University Hospital of Brussels and was carried out in accordance with the ethical principles for medical research involving human subjects established by the Declaration of Helsinki, protecting the privacy of all participants as well as the confidentiality of their personal information. All data were fully anonymized. The need for consent in this study was waived by the ethical committee.

### Statistical Analysis

Continuous variables were presented as means with standard deviations (SD) or median [interquartile range (IQR)] for skewed variables. Categorical variables were expressed as percentages. Normality of data was tested using Kolmogorov–Smirnov test. Comparisons of continuous variables were done using Student *t*-test or Mann–Whitney *U*-test and of binominal variables using chi-square or Fisher exact test, respectively. Intraobserver and interobserver variability for CAC score assessment was tested by Cohen's Kappa coefficient. The following criteria for Kappa coefficient were used to interpret the results: <0.00 = poor, 0.00–0.20 = light, 0.21–0.40 = fair, 0.41–0.60 = moderate, 0.61–0.80 = substantial, and 0.81–0.99 = almost perfect (11). Univariate and multivariate logistic regression models were used to evaluate potential predictors of MACE. Variables included in the multivariate analysis were chosen based on their statistical significance in the univariate analysis (*p* < 0.05) and on their clinical significance. Specificity, sensitivity, and negative predictive value of CAC score = 0 were calculated using a cross-tabulation table. Specificity was defined as the probability that a test result will be negative when the disease is not present (true negative rate). Sensitivity was defined as the probability that a test result will be positive when the disease is present (true positive rate). Negative predictive value was defined as the probability that the disease is not present when the test is negative (12, 13). Statistical significance was considered for a *p* < 0.05. Statistical analyses were performed using IBM SPSS Statistic for Windows, Version 27.0 (IBM Corp., Armonk, NY, USA).

## Results

A total of 280 patients (63.2 ± 16.7 years old, 57.5% male) were included in the analysis.

Mean length of hospitalization was 13.6 ± 13.2 days. Sixty-one patients (21.8%) had at least one MACE: 16 (5.7%) patients presented acute heart failure, 15 (5.3%) patients had atrial fibrillation, 4 (1.4%) patients presented acute coronary syndrome [2 (0.7%) patients had a non-ST elevation myocardial infarction and 2 (0.7%) patients had unstable angina], and 3 (1.0%) patients presented a stroke, respectively. In-hospital mortality rate was 16.1% (45 patients). CAC score = 0 was found in 46.7% (131) patients, vs. 53.2% (149) patients with CAC score ≥1. The baseline characteristics of the study population and the comparison between patients with a CAC score = 0 and CAC score ≥ 1 are shown in Table 1.

**Table 1:** Comparison between patients with CAC score 0 and those with CAC score ≥1.

	**Total (*n* = 280)**	**CAC score = 0 (*n* = 131)**	**CAC score ≥ 1 (*n* = 149)**	***p*-value**
Age (years)	63.2 ± 16.7	53.7 ± 13.1	72.7 ± 13.2	<0.001
Weight (kg)	80.5 ± 16.7	84.4 ± 16.3	76.6 ± 15.8	<0.001
BMI (kg/m^2^)	27.8 ± 5.2	28.9 ± 5.2	26.8 ± 4.9	0.001
Male gender (*n*, %)	161 (57.5)	76 (58.0)	76 (60.3)	0.707
**History**				
Heart failure (*n*, %)	6 (2.1)	1 (0.8)	5 (4.0)	0.089
Valve disease (*n*, %)	6 (2.1)	1 (0.8)	5 (4.0)	0.089
Atrial fibrillation (*n*, %)	14 (5.0)	3 (2.2)	11 (7.3)	0.023
CKD (*n*, %)	32 (11.4)	9 (6.9)	23 (18.3)	0.006
Chronic pulmonary disease (*n*, %)	43 (15.3)	20 (15.2)	23 (15.4)	0.718
Cancer (*n*, %)	28 (10)	7 (5.3)	21 (16.7)	0.004
**Risk factors**				
Hypertension (*n*, %)	128 (45.7)	40 (30.5)	78 (61.9)	<0.001
DM (*n*, %)	64 (22.8)	33 (25.2)	31 (24.6)	0.913
Dyslipidemia (*n*, %)	89 (31.8)	39 (29.8)	50 (39.7)	0.095
Smoking (*n*, %)	30 (10.7)	13 (9.9)	17 (13.5)	0.373
**Laboratory values**				
Hemoglobin (g/dl)	13.5 ± 1.8	13.7 ± 1.7	13.4 ± 2.0	0.205
Platelets (10^3^/mm^3^)	218.4 ± 86.8	218.3 ± 84.1	212.2 ± 86.5	0.572
WBC (10^3^/mm^3^)	7.8 ± 4.4	7.2 ± 3.0	8.1 ± 5.0	0.072
CRP (mg/L)	135.7 ± 96.5	128.0 ± 97.9	140.1. 94.1	0.312
D-dimers (ng/ml)	1,638.6 ± 2,720.7	1,048.5 ± 1,442.4	1,830.9 ± 2,613.0	0.042
LDH (U/L)	968.4 ± 1,183.8	988.6 ± 608.8	1,000.3 ± 595.8	0.877
Lactate (mmol/L)	1.0 ± 0.6	1.0 ± 0.5	1.1 ± 0.6	0.659
cTnT (μg/L)	0.02 ± 0.04	0.01 ± 0.01	0.02 ± 0.01	<0.001
Creatinine (mg/dl)	1.2 ± 1.2	1.0 ± 0.9	1.3 ± 1.4	0.012
**Chest CT**				
Ground-glass opacity (*n*, %)	226 (80.7)	115 (87.7)	111 (74.4)	0.854
Interlobular septal thickening (*n*, %)	25 (8.9)	9 (6.8)	17 (11.4)	0.076
Pulmonary consolidation (*n*, %)	94 (33.5)	44 (33.5)	50 (33.5)	0.267
Pleural effusion (*n*, %)	14 (5.0)	4 (3.0)	10 (6.7)	0.068
ICU admission (*n*, %)	71 (18.2)	33 (25.1)	39 (26.1)	0.500

*BMI, body mass index; CKD, chronic kidney disease; DM, diabetes mellitus; WBC, white blood cells; NLR, neutrophil to lymphocyte ratio; CRP, C-reactive protein; LDH, lactate dehydrogenase; cTnT, cardiac troponin T; CT, computed tomography; ICU, intensive care unit; CAC, coronary artery calcium*.

Univariate analysis for the prediction of MACE is shown in Supplementary Table 1.

Multivariate analysis (Table 2) showed that a CAC score of 0 was inversely associated with the occurrence of MACE [*p* = 0.027, odds ratio (OR) = 0.209, 95% confidence interval (CI) 0.052–0.833]. The negative predictive value of CAC score for MACE was 84.5% (sensitivity 72%, specificity 55%).

**Table 2:** Predictors of MACE.

	**OR**	**95% CI**	***p***
Age	1.067	1.009–1.129	0.024
Male gender	0.702	0.221–2.228	0.548
Atrial fibrillation	1.175	0.182–7.595	0.865
Creatinine	1.018	0.49–2.090	0.962
CRP	1.009	1.004–1.015	0.001
cTnT	1.072	1.026–1.120	0.002
CAC score = 0	0.209	0.052–0.833	0.027

*CRP, C-reactive protein; cTnT, cardiac troponin T; CAC, coronary artery calcium; OR, odds ratio; CI, confidence interval; MACE, major adverse cardiovascular events*.

Reproducibility of CAC score assessment using Cohen's k showed substantial intraobserver and interobserver agreement for the total CAC score assessment (*k* = 0.859, 95% CI 0.678–1.000, *p* < 0.001 and *k* = 0.795, 95% CI 0.581–1.000, *p* < 0.001, respectively).

## Discussion

The main findings of this study were the following: (1) MACE rate in COVID-19 hospitalized patients was 21.8%; (2) the absence of CAC was independently associated with a lower rate of MACE in COVID-19 hospitalized patients.

COVID-19 promotes a rapid systemic inflammation and cytokine storm, which can cause vascular dysfunction, destabilization of atherosclerotic plaques, or myocardial infiltration, which are potential pathways for cardiovascular complications (14). The most commonly reported MACE in COVID-19 hospitalized patients include heart failure, arrhythmia, and acute coronary syndrome, similar to results from the present study (14–16). Moreover, patients with pre-existing cardiac disease are more predisposed to develop cardiac complications during hospitalization for COVID-19 (14, 15).

Similar to previous reports, in the present study, older age was independently associated with worse outcome of COVID-19 patients (17, 18). Moreover, an increased cardiac troponin independently predicted MACE, which is in line with recent studies showing evidence of myocardial injury in hospitalized COVID-19 patients and subsequently increased disease severity (2, 19).

Current guidelines consider CAC score to be a risk modifier in primary prevention of cardiovascular disease (5, 6). Moreover, CAC score has been shown to improve cardiovascular risk prediction in addition to classical risk factors (5, 6, 20) and to be a potential tool for risk reclassification (21–24). The Multi-Ethnic Study of Atherosclerosis (MESA) showed that CAC improved risk prediction at 10-year follow-up compared with traditional risk factors alone (25).

Interestingly, multiple studies have focused on the role of the absence of CAC as a potential downward cardiovascular risk reclassification (26–29). In the present study, the absence of CAC score independently predicted lower MACE rate in COVID-19 hospitalized patients.

Dillinger et al. (30) evaluated the role of CAC in COVID-19 patients hospitalized at the intensive care unit (ICU) and showed that the presence of CAC score was associated with the occurrence of mechanical ventilation, extracorporeal membrane oxygenation, or death. Compared to the present study and other previous series, mortality rate in the study of Dillinger et al. (30) was significantly lower, even if the authors reported only the mortality among ICU patients (2, 19, 31, 32). In our cohort, 71 (25.7%) patients were transferred to ICU, among whom 19 (26.7%) died. Surprisingly, the proportion of elevated CAC score in patients younger than 61 years old was higher in the study of Dillinger et al. (30) compared to the results from our cohort. For the same group of ethnicity, the MESA study showed that CAC score increased with age, which is comparable to data from the present study (33). In contrast to MESA, there was no significant difference in CAC score between genders in this study.

In another recent report by Nai Fovino et al. the presence of CAC in COVID-19 patients was associated with ICU admission and in-hospital mortality (34). However, this study had a small sample population in whom CAC score was evaluated as high or low-intermediate, and potential confounders were not included; therefore, the results cannot be compared to our cohort. Zimmerman et al. also evaluated the role of CAC in the prediction of ICU admission and death in COVID-19 patients (35). Nevertheless, in this study, patients with a history of coronary artery disease were not excluded from the analysis, and the potential relationship between CAC and inflammatory markers was not assessed.

Although recent studies focused on the power of CAC score 0 to predict an improved cardiovascular outcome, data regarding the role of CAC in COVID-19 patients with classical cardiac risk factors are still limited (27, 36). In this study, the absence of CAC translated into a low risk for MACE in COVID-19 patients, independent of age and the presence of risk factors or inflammation, reinforcing the idea that the assessment of CAC score in hospitalized COVID-19 patients could be a useful marker for patients' risk stratification and management.

At the beginning of the pandemic, RT-PCR tests were not widely available; therefore, a systematic chest CT was performed in almost all COVID-19 patients. The ability to assess CAC score on non-gated chest CT allows the application of CAC to the risk evaluation of COVID-19 patients with no additional cost or time consumption. Moreover, most studies report a low-dose radiation for chest CT in COVID-19 patients (37). The semi-qualitative assessment of CAC on routine chest CT has proved to be accurate and reproducible when compared to Agatston scoring (10). Similarly, in our study intraobserver and interobserver reproducibility of CAC score was very good.

Evidence that viral infections represent a trigger for cardiovascular events is increasing, but data regarding long-term follow-up of patients admitted with respiratory viral diseases are still scarce (38, 39). Future directions should focus on the implementation of CAC score into mid-term and long-term follow-up of this particular population, to provide a more precise and earlier estimation of cardiovascular risk.

## Study Limitations

This was a single-center study, the sample size was relatively small, and no comparison with a control group was performed; therefore, the extrapolation of these results is limited. The method used to assess CAC is a semi-qualitative scoring system using a non-gated chest CT. The absence of triggering, the lower temporal resolution, and larger field of view which alters the voxel size might modify CAC score assessment. However, this method has been previously validated against quantitative CAC assessment, and its accuracy to predict Agatston score was demonstrated (10).

## Conclusion

In this study, the absence of CAC had a high negative predictive value for MACE in patients hospitalized with COVID-19, independent of the presence of cardiac risk factors. A semi-qualitative assessment of CAC is a simple, reproducible, and non-invasive measure that may be useful for the risk stratification of COVID-19 patients.

## Data Availability Statement

The raw data supporting the conclusions of this article will be made available by the authors, without undue reservation.

## Ethics Statement

The studies involving human participants were reviewed and approved by Medical Ethics Committee, University Hospital of Brussels. Written informed consent for participation was not required for this study in accordance with the national legislation and the institutional requirements.

## Author Contributions

M-LL, SL, AM, and BC contributed to the conception and design of the study. M-LL, AM, and SL wrote the first draft of the manuscript. AM and JMa performed the statistical analysis. DB, JMe, SB, and KT performed CT analysis. ES, BR, KV, TD, BK, XG, and CF participated to the investigation, clinical assessment and database. CW, SD, and BC contributed to project administration, supervision and validation. All authors contributed to manuscript revision, read, and approved the submitted version.

## Conflict of Interest

The authors declare that the research was conducted in the absence of any commercial or financial relationships that could be construed as a potential conflict of interest.
