# Supplementary material for: Prognostic Value of Coronary Artery Calcium Score in Hospitalized COVID-19 Patients
Source: Front Cardiovasc Med. 2021 Jul 9;8:684528. doi: 10.3389/fcvm.2021.684528 (PMC8301217; doi:10.3389/fcvm.2021.684528)
Supplement: Supplementary file 1 [file Table_1.docx]

Supplementary Material

# Supplementary Table 1

**Univariate analysis for the prediction of major adverse cardiovascular events (MACE)**

|  | OR | 95% CI | P |
| --- | --- | --- | --- |
| Age | 1.066 | 1.043 - 1.090 | 0.001 |
| Male gender | 0.511 | 0.288 - 0.908 | 0.022 |
| Obesity | 0.903 | 0.481 - 1.697 | 0.752 |
| Hypertension | 1.121 | 0.634 - 1.91 | 0.694 |
| Diabetes mellitus | 1.196 | 0.631 - 2.267 | 0.584 |
| Dyslipidemia | 0.974 | 0.536 - 1.770 | 0.931 |
| Atrial fibrillation | 3.889 | 1.308 - 11.561 | 0.015 |
| Chronic pulmonary disease | 1.510 | 0.738 - 3.090 | 0.259 |
| Chronic kidney disease | 1.540 | 0.715 - 3.317 | 0.270 |
| Cancer | 1.338 | 0.564 - 3.175 | 0.509 |
| CRP | 1.005 | 1.002 – 1.008 | 0.001 |
| cTnT | 1.077 | 1.044 – 1.110 | 0.001 |
| D - dimers | 1.000 | 1.000 – 1.001 | 0.096 |
| Creatinine | 1.254 | 1.022 - 1.538 | 0.030 |
| CAC score = 0 | 0.477 | 0.258 - 0.883 | 0.019 |

CRP: C – reactive protein; cTnT: cardiac troponin T; CAC: coronary artery calcium; OR = odds ratio; CI = confidence interval
